# Molecular and Clinical Significance of PDGFR Family in Papillary Thyroid Cancer: A Systematic Review

**DOI:** 10.34172/apb.46975

**Published:** 2026-05-24

**Authors:** Atiyeh Dabouian, Roya Ghafoury, Mahboobeh Nazari, Omolbanin Asadi Ghadikolaei, Mohammad E. Khamseh, Sara Cheraghi

**Affiliations:** ^1^Endocrine Research Center, Institute of Endocrinology and Metabolism, Iran University of Medical Sciences, Tehran, Iran; ^2^Monoclonal Antibody Research Center, Avicenna Research Institute, ACECR, Tehran, Iran; ^3^Nutrition and Health Research Center, Nasim Institute, Tehran, Iran

**Keywords:** PDGFRα/β, Thyroid cancer, Biomarker, PDGFR expression, Genetic variation

## Abstract

**Purpose:**

Papillary thyroid cancer (PTC) is the most prevalent endocrine cancer. Activation of platelet-derived growth factor receptors (PDGFRs) is involved in the development of thyroid cancers. This systematic review aims to evaluate the molecular and clinical significance of PDGFRs in PTC.

**Methods:**

A comprehensive literature search was performed in PubMed, Scopus, and Web of Science databases up to August 2024, focusing on studies evaluating PDGFRα/β variants, expression levels, and clinical outcomes in PTC. Eligible studies were selected according to PRISMA guideline. Studies including unknown histology, insufficient data, case reports, non-human experiments, and review article were excluded. The REMARK criteria were applied for quality assessment of the included studies.

**Results:**

A total of twelve studies were included. Genetic alterations in the promoter of PDGFRα (rs6554162 and rs1800812) showed a significant association with increased risk of PTC, while synonymous variants were more frequently detected in benign tumors. In contrast, noncoding variants in PDGFRβ showed no association with PTC. Overexpression of PDGFRα was significantly associated with lymph node metastasis, larger tumor size, poorer disease-free survival (DFS) with some inconsistency and poor response to radioactive iodine therapy. Findings on PDGFRβ expression were inconsistent. However, one study reported its upregulation in metastatic PTC and its association with shorter overall survival in patients with BRAFV600E.

**Conclusion:**

PDGFRα may serve as a potential biomarker associated with aggressive tumor behavior and treatment failure in people with PTC. Further experimental studies are needed to validate the results. Targeting PDGFRα may represent a therapeutic approach, but evidence is still insufficient.

## Introduction

 Thyroid carcinoma (TC) is a global health issue and is the most prevalent cancer of the endocrine system,^1^ with a higher incidence in females.^2^ Differentiated TC (DTC) accounts for more than 90% of TC and includes papillary, follicular and poorly differentiated TC.^3^ Among these, Papillary thyroid carcinoma (PTC) is the most prevalent, with an incidence rate more than 80%.^4^ The recent evidence suggests the period from 1990 to 2019 an annual increase in the TC occurrence, additionally, projections for the period 2020 to 2030 suggest an ascending trend in TC burden.^5^ PTC has several histological subtypes: the most common is the classical type with 50% prevalence. Other subtypes include follicular variant, tall cell, columnar cell, hobnail, diffuse sclerosing, solid/trabecular, and clear-cell variants.^6,7^ These subtypes display different biological behavior such as aggressiveness.^8^ Environmental risk factors, molecular and epigenetic alterations can influence the incidence and aggressiveness of TC, as well as impact the response to the treatment.^9,10^ Platelet-derived growth factors receptors (PDGFRs) are expressed in various malignant cells. PDGFRs are members of receptor tyrosine kinase (RTK) type III which binds to an intracellular RTK through an extracellular ligand. Activation of PDGFR signaling pathways stimulates downstream cascades such as (MAPK/ERK, PI3K/AKT, and JAK/STAT) which collectively promote the tumor cell proliferation, angiogenesis, invasion, and metastasis.^11,12^ PDGFR alpha ((PDGFRα) is typically found at low levels in normal tissues, but its expression is significantly elevated in tumor cells. Additionally, overexpression of PDGFRα has been observed in metastatic, recurrence, and treatment resistance in PTC. In contrast, PDGFRβ is frequently explored in various tissues and emerges as a mediator in angiogenesis.^13-15^ however, its overexpression has been observed in anaplastic thyroid carcinomas (ATC).^16^ Generally, the expression of PDGFRs is associated with tumor cell growth and response to therapy.^17^ There is conflicting evidence concerning the prognostic significance of PDGFRs, and the potential benefit as targeted therapies. This underscores the necessity for a comprehensive systematic review of the current studies. In this systematic review we evaluate the clinical significance of PDGFRs in papillary thyroid cancer.

## Method and Materials

###  Search Strategy

 The relevant studies were obtained in PubMed, Scopus, and Web of Science (WoS) databases through a systematic search that publish up to August 2024.The MeSh terms “thyroid Cancers” AND “PDGFR” AND “mutation” was used as search algorithms without any language restriction and also the search strategy was restricted to titles/abstracts (Table S1). The recent version of PRISMA 2020 guideline was performed in line in this study.^18^

###  Selection Criteria

 The relevant studies were selected according to the following inclusion and exclusion criteria. The inclusion criteria included: investigated the expression or assay of PDGFRs in patients with TCs, human studies, and available studies in English language. The ineligible studies were excluded based on the following criteria: studies with undetermined histology, case reports, *in vitro* and animal experiments, review, meeting abstracts, letters, insufficient data, and using duplicate data.

###  Data Extraction 

 All search articles were imported into the EndNote management software. The title, abstract screening and full-text assessment were performed by one reviewer (AD), the second evaluator (SCh) cross check the screening and included data extraction. Differences between reviewers were resolved through discussion. Data extraction was carried out by focusing the following data: article features, ethnicity, number of patients and control, age, sex, thyroid cancer histology, tumor size, stage of tumor, expression in case and control groups, lymph node and distant metastasis, tumor recurrence, invasion, extra-thyroid extension, death, overall survival (OS), and disease-free survival (DFS).

###  Quality Assessment

 The quality of the studies was assessed independently by AD and SCh for all included studies. The REMARK (Reporting Recommendations for Tumor Marker Prognostic Studies) criteria were to evaluate included references as the primary tool.^19^ This assessment includes twenty criteria classified into four main items: introduction, methods, results, and discussion. Each item was scored 1 if fully reported, and a score of 0.5 is used for criteria that are not fully filled. The overall quality was categorized as low (≤ 10), medium (≤ 15), and high (> 15). Any discrepancies between reviewers were resolved through discussion.

###  Data Synthesis

 Due to heterogeneity in outcome reporting and extractable effect sizes, meta- analysis was not feasible. Therefore, the findings were synthesized using a systematic descriptive approach.

## Results

###  Study Selection

 A total of 819 articles were included in the search strategy after removing duplicate articles. In the first step, 676 records were excluded by screening the title and followed by abstract screening and full text assessment, 141 irrelevant articles were removed. Finally, 12 eligible studies were included in the systematic review. The PRISMA flow diagram illustrating the study process is presented in Figure 1.

**Figure 1:**
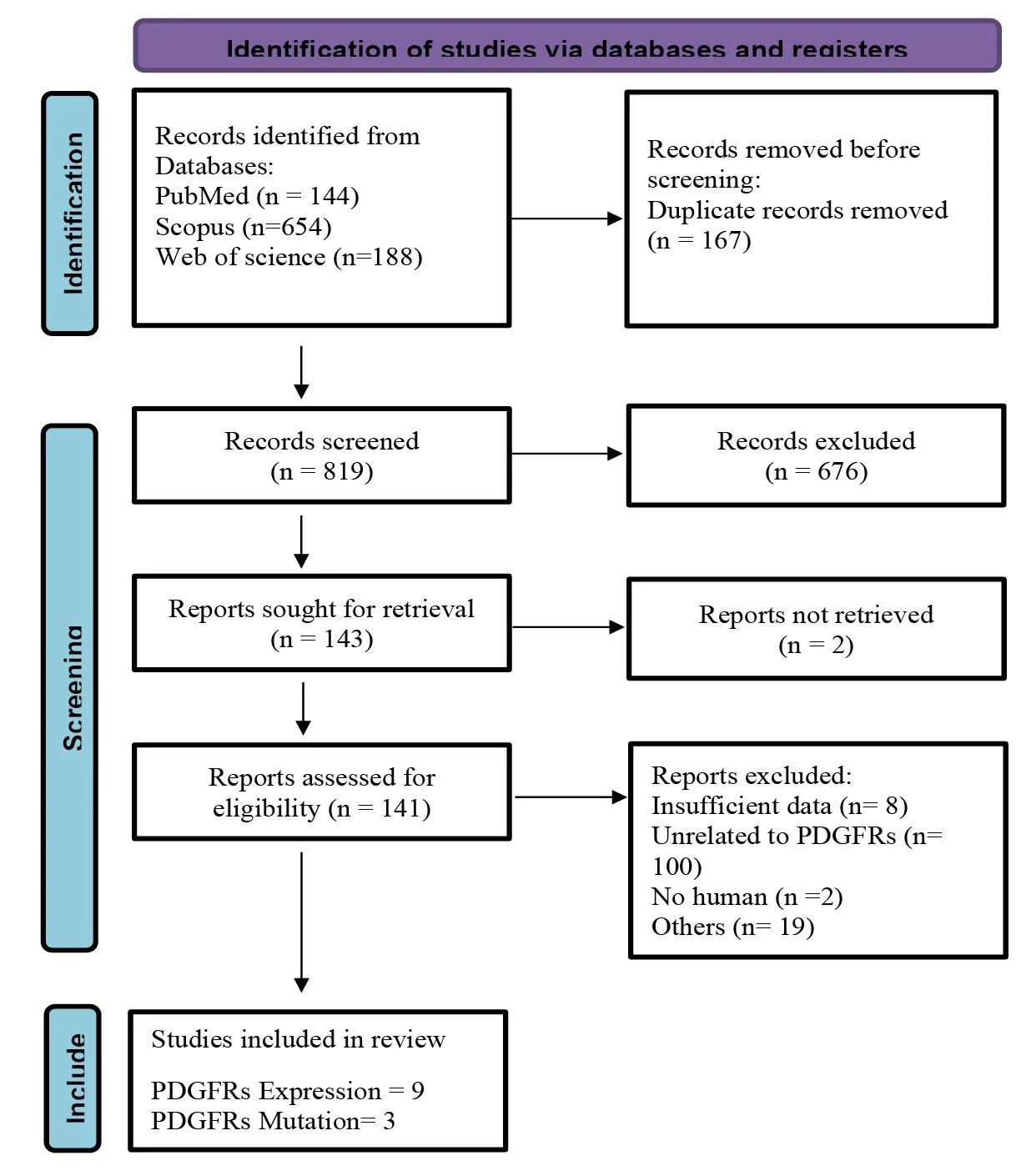
PRISMA flowchart for study selection

###  Quality Assessment of Included Studies 

 The result of REMARK criteria showed a mean quality score of 15.58 points (range 12-18). Individual study scores are presented in Table S2. All studies achieved scores above 10, indicating medium to high-quality quality. The nine studies had a score ≤ 15, while three studies exceeded a score of 15. Analysis of REMARK items indicated that specimen characteristics were under-reported across some studies. Clinical endpoints, multivariate analyses, estimated effects, and further investigations were also frequently incompletely described. In contrast, study design, assay methods, variables examined, statistical approaches, and result interpretation was generally well reported, highlighting areas for improvement in reporting quality.

###  Description of Studies

 We included the nine gene expression studies and three genetic alteration studies with a total of 1,350 and 347 patients, respectively. Additionally, PDGFRα was assessed more frequently than PDGFRβ.The Immunohistochemistry (IHC) technique was the most commonly used method to assess PDGFRs expression; however, some studies used RT-qPCR or western blotting. The variants identification was mainly performed through sequencing methods.

###  Molecular and Clinical Characteristics of PDGFRα Status 

 Most included studies evaluated the relationship between PDGFRα molecular and clinicalfeatures and clinicopathological features of PTC. The assessed outcomes included tumor size, lymph node and distant metastasis, extrathyroidal extension, and invasion parameters. Table 1 and 2 summarize the molecular patterns and clinical features of PDGFRs in the included studies.

**Table 1:** Genetic alterations of PDGFRα and PDGFRβ in papillary thyroid cancers.

**Study**	**PDGFRs/** **Tumor Type**	**Alternation Origin**	**Method**	**Genetic Alteration**	**Key Findings**
Cruz-Romero et al. (2024)^20^	PDGFRα/PTC	Somatic	NGS	CNVs	Higher frequency of variants was reported in classical and tall cell PTC. Highest SNP was detected in BRAF. BRAF^V600E^ showed the most prevalent in classic PTC.
Borowczyk et al. (2019)^21^	PDGFRα/PTC	Somatic	NGS	rs1873778 rs2228230	More frequent in benign nodules than malignant samples.
Kim et al. (2012)^22^	PDGFRα/PTC	Germline	Sanger	rs6554162rs1800812	The variants of PDGFRα were associated with increased PTC risk in haplotype analysis (*P* < 0.05)
	PDGFRβ/PTC	Germline	Sanger	rs3828610	The variant of PDGFRβ showed no significant association with PTC risk.

CNV: Copy Number Variation, NGS: Next-generation sequencing, PTC: Papillary Thyroid Cancers, SNP: Single Nucleotide Polymorphism

**Table 2:** The PDGFRs gene expression in thyroid cancer.

**Study**	**Clinical Data**	**Results**
Zhu et al. (2023)^23^	125 PTC (36 M/89 F) Age: 24-77 years Extrathyroidal invasion: 27 AJCC Staging: I = 114, II = 10, III = 1 101 with BRAF^V600E^ Receptor: PDGFRα Method: IHC	- CAFs markers: PDGFRα, α-SMA, FAP, and Vimentin were evaluated - α-SMA and FAP were significantly associated with tumor size, LNM, and extrathyroidal invasion with *P* < 0.0001 in PTC, BRAF^V600E^ co-occurred with higher α-SMA/FAP level (*P* < 0.05) - α-SMA and FAP introduced as CAFs markers - Vimentin positivity was in tumor cells, thyroid normal cell, and stroma
Shi et al. (2020)^24^	90 PTC (29M/61 F); 23 Normal tissue Mean Age: 42.6 Lymph Node Stage: N0 = 68, N1 = 22 Tumor Stage: T1-T2 = 61, T3-T4 = 29 Receptor: PDGFRα Method: IHC	-High levels of PDGFRα were observed in 50 PTC patients and signiﬁcantly association when compared with non-tumor tissues (*P* < 0.001) -Overexpression of PDGFRα was related with older age (*P* < 0.001) and advanced tumor stage (*P* = 0.019) - No significant associated was found with LNM and sex (*P* = 0.078) -Patients with higher PDGFRα showed poor OS (*P* = 0.02) -DFS showed no significant difference (*P* = 0.3)
Adewuyi et al. (2018)^25^	162 PTC (26 M/136 F); 59 Node Positive Mean Age: > 45 Receptor: PDGFRα Method: IHC	-Expression of PDGFRα was just correlated with PDGFA ligand (OR = 4.6, *P* = 0.004) -Moderate or strong levels of PDGFA had recur potential -PDGFA expression associated with metastasis (*P* = 0.0001) - PDGFRα was not prognostic for OS/DFS; high level of PDGFA expression has prognostic effect (DFS with *P* = 0.0004)
Sun et al. (2016)^26^	339 PTC (303 Classic, 36 FVPTC), (67M/272 F) Age: < 45 = 153, ≥ 45 = 186 238 with BRAF^V600E^ Receptor: PDGFRα/β Method: IHC	-Overexpression of PDGFRα was more pronounced in classic type compared to the follicular type (*P* < 0.001) - PDGFRα expression was higher in tumor cell with BRAF^V600E^ (*P* < 0.001) - BRAF^V600E^ co-occurred with higher stromal PDGFRα/β than BRAF wild tumor (PDGFRα: *P* = 0.002, PDGFRβ: *P* < 0.001) - High stromal PDGFRβ predicted shorter OS (*P* = 0.03) - PDGFRα expression was higher in tumor vs stromal (*P* = 0.004)
Campistrous et al. (2016)^15^	181 PTC: 68 with LNM, 113 without LNM (35 M/146 F) Mean Age: 45.9 Receptor: PDGFRα Method: IHC	-Overexpression of PDGFRα and low level of TTF1 was found in metastatic tumors (*P* = 0.005) -Primary tumor without metastasis were lack of PDGFRα -The level of NIS was decreased in metastatic PTC cases in the presence of PDGFRα -Iodine dose therapy was higher in PDGFRα positive tumor (*P* = 0.005) -PDGFRα-positive tumor had more potential to recurrence (*P* < 0.0001)
Lee et al. (2016)^27^	177 PTC (31 M/146 F) Mean Age: > 42 Receptor: PDGFRβ TNM Stage: I-II = 84, III-IV = 93 Method: IHC	- LETM1 overexpression correlated with LNM and LVI patients (OR = 2.3, OR = 4.7) -Western blot analyses showed increased PDGFB and LETM1 expression in stage T3 tissue samples - LETM1 inhibition reduced PDGFB- PDGFRβ signaling
Zhang et al. (2012)^13^	124 PTC; 58 with and 66 without node metastasis Age: No data Receptor: PDGFRα/β Method: IHC	- PDGFRα was overexpressed in the primary tumors with LNM (83%) (*P* < 0.0001) - PDGFRβ expression was common in the tumors cell vs benign nodule (*P* < 0.0001)
Durante et al. (2011)^28^	90 PTC (25 M/65 F) Median Age: 46 years 55 with BRAF^V600E^ Receptor: PDGFRβ Method: IHC/qRT-PCR	- PDGFRβ mRNA levels was lower in BRAF^V600E^ tumors
Elliott et al. (2008)^29^	31 ATC; 7 PTC (15M/16 F) Mean Age: 60.8 Receptor: PDGFRβ Method: IHC	-Overexpression of PDGFRβ was exhibited in 16% of ATC patients -Overexpression of PDGFRβ was exhibited in 14% of PTC patients

AJCC: American Joint Committee on Cancer, IHC: Immunohistochemistry, LNM: Lymph Node Metastasis, LVI: Lymph Vascular Invasion, M: Male, F: Female

 Somatic mutations in PDGFRs genes were uncommon; however, three studies evaluated PDGFRα genetic alterations (Table 1). Two case and control studies of the Korean populations with PTC, single nucleotide polymorphisms (SNPs) in the promoter region of both PDGFRα and PDGFRβ were assessed in germline. The SNPs rs6554162 and rs1800812 of PDGFRα were genotyped in 93 cases and 212 healthy individuals, showing a significant association with increased risk for PTC, particularly in the dominant model (aa + Aa vs AA), with p-values of 0.0005 and 0.007, respectively. These promoter-region SNPs may impact both transcription and translation of PDGFRα protein. Genotype labels “A” and “a” are symbolic representations and do not indicate any SNP. Furthermore, the allele “a” is classified as a risk allele.^22^ Additionally, two synonymous PDGFRα variants, rs1873778, and rs2228230, were identified in FNA (fine needle aspiration) specimens using NGS (Next-Generation Sequencing). These variants were detected in five benign nodules and only one malignant sample. Notably, there were significantly more variants of PDGFRα in the benign nodules compared to the malignant one. The most prevalent mutations in malignant tumors were detected in the RET and KDR genes rather than PDGFRα.^21^

 In a Colombian cohort, 14 PDGFRα copy-number variations (CNVs) (7.53%) were found in different subtypes of PTC with most frequency in classic (n = 9) and tall cell (n = 2). Additionally, a lower rate of CNVs was occurred in the KIT gene (1.6%). Several pathogenic variants (in BRAF, RAS, PIK3CA, and IDH1 genes) were also detected, but no association were observed with clinicopathological features.^20^

 Five of eight studies reported a significant association between PDGFRα overexpression and PTC.^13,15,24-26^ Among these, three studies demonstrated a considerable association between PDGFRα expression and lymph node metastasis in PTC patients (*P* < 0.05).^13,15,25^

 Campistrous et al. and Zhang et al. exhibited markedly higher node positive tumors in PDGFRα-positive tumor (*P* < 0.0001). Adewuyi et al. demonstrated the specificity and sensitivity of PDGFRα expression in both node-negative and node-positive PTC, with values of 71% and 86% respectively. Also the expression of PDGFA in nodal positive was strongly associated with a p-value lower than 0.0001.^13,15,25^ Unlike, in Shi et al. study, there was no correlation observed between the expression of PDGFA and lymph node metastasis.^24^

 Additionally, high levels of PDGFRα expression have been observed to promote invasion in PTC patients and also cell line.^13,24^ In the study by Campistrous et al, a significant relationship showed between the upregulation of PDGFRα and larger tumor sizes. They also observed that patients with PTC and high levels of PDGFRα had a higher rate of recurrence ^15^. Invasion was also reported to relate with PDGFRα expression in just one study.^24^ In contrast, the study by Zhu et al, showed poor expression of PDGFRα, and no relation was observed.^23^

 The prognostic value of PDGFRα was evaluated in three included studies, which provided conflicting prognostic evidence. The study by Adewuyi et al. examined PDGFRα and its PDGFA expression to evaluate their association with disease-free survival (DFS).The intense expression showed a significant association with DFS (*P* = 0.0004), indicating an increased recurrence rate in PTC patients.^25^ Specifically, upregulation of PDGFRα was significantly correlated with higher recurrence rates within five years in patients with PTC compared with low PDGFRα expression levels (*P* < 0.0001) ^15^. Unlike the Shi et al. study, no significant difference in DFS was demonstrated between high level of PDGFRα expression and its absence in patient with PTC.^24^

 Two studies explored therapeutic implications of PDGFRα. Campistrous et al. compared iodine therapy outcomes in patients with high PDGFRα expression to those with lacking of PDGFRα expression. They found that upregulation of PDGFRα decreased the NaI symporter (NIS) levels, reducing iodine uptake and contributing to treatment resistance, leading to the administration of higher iodine doses (*P* = 0.005). This finding suggests that PDGFRα may serve as a potential predictive marker for radioactive iodine (RAI) therapy.^15^ Moreover, PDGFRα activation enhanced downstream signaling pathways (MAPK/ERK and PI3K/Akt pathways), leading to cellular proliferation. Zhang et al. showed that sunitinib inhibits PDGFRα activation and reduces the activity of these downstream pathways, potentially offering a targeted therapeutic approach.^13^

###  Molecular and Clinical Characteristics of PDGFRβ Status 

 Five studies evaluated PDGFRβ variants and their association with clinicopathological feature in PTC. The molecular and clinical findings related to PDGFRβ are summarized in Table 1 and 2.

 In PTC tumor cells, the variant rs3828610 in PDGFRβ was genotyped in 305 patients and healthy individuals. This SNP showed no significant association with PTC risk, no correlation was observed between rs3828610 and tumor size, TNM stage, and extrathyroidal extension.^22^ However, low level of PDGFRβ was observed in patients with PTC in the presence of BRAF^V600E^.^28^ The expression of PDGFRβ was analyzed in the presence of RET, RAS, BRAF, and LETM1 mutations. One small study evaluated PDGFRβ expression independently in ATC and seven PTC samples and found no significant clinicopathological association (*P* = 0.3).^29^

 Metastatic TCs is a critical factor in the progression of tumors cells. The role of PDGFRβ in metastasis remains unclear. Two studies explored the association between PDGFRβ expression and metastatic manner. Higher PDGFRβ mRNA levels were associated with metastatic and invasive behavior in PTC, co-expressed with LETM1.^27^ Furthermore, other cohort showed no significant difference in PDGFRβ expression between node- positive and node-negative PTC tumors.^13^

 There is inconsistent evidence regarding the prognostic value of PDGFRβ expression in PTC. No significant association was observed between PDGFRβ expression and DFS in PTC samples. However, high levels of PDGFRβ expression correlated with shorter OS in BRAF^V600E^ positive PTC patients.^26^

## Discussion

 In this systematic review included 1,295 patients with PTC across nine expression studies, alongside three additional genetic alteration studies involving 347 patients. The authors explored the distinct molecular and clinical roles of PDGFRα and PDGFRβ in PTC. PDGFRα emerges as a consistent molecular driver in PTC, with potential clinical implications due to its strong association with lymph node metastasis, larger tumor size, invasion, and recurrence. These associations are supported by both protein expression data and germline single nucleotide polymorphisms (SNPs). Conversely, PDGFRβ exhibits a heterogeneous expression pattern influenced by tumor histology and mutational context, such as BRAF^V600E^ status, with its prognostic and therapeutic significance remaining less well defined due to limited and conflicting evidence. Integration of genetic, transcriptomic, and clinical findings highlights the importance of comprehensive molecular profiling in clinical assessment and positions PDGFRα as a robust biomarker and promising therapeutic target in thyroid cancer, while underscoring the need for further research on PDGFRβ. These insights contribute to improved risk stratification and the advancement of precision-guided therapeutic strategies in thyroid cancer management.

 The quality and risk of bias of the included studies were systematically evaluated using the REMARK criteria, which are designed specifically for tumor marker prognostic studies.^19^ The overall mean quality score was 15.58 (range 12–18), indicating that all studies met a medium to high-quality threshold. This suggests a generally robust methodological approach across the selected studies. However, some limitations were noted, including variability in study design, sample size, and reporting completeness, which may contribute to potential biases. For instance, differences in the techniques used for PDGFR assessment (e.g., immunohistochemistry versus molecular methods, differences in IHC scoring systems, inconsistent cut-off definitions, and the specific cellular compartment evaluated (tumor cells versus stromal expression). In addition, some studies assessed related ligands such as PDGFA rather than PDGFRα itself, which may further contribute to inconsistent findings. Variations in outcome definitions, such as DFS versus recurrence, may also influence the comparability of results.), inconsistent reporting of clinical variables, and heterogeneity in patient populations may have affected the comparability and generalizability of findings. Moreover, the relatively small number of studies and their retrospective nature could introduce selection and reporting biases. Despite these limitations, the quality assessment supports the reliability of the evidence synthesized in this review, though results should be interpreted with caution given the identified sources of potential bias.

 The molecular data from our systematic review reinforce the oncogenic role of PDGFRα in PTC, consistent with its well-established function as a receptor tyrosine kinase that activates canonical proliferative and survival pathways. Two SNPs located in the PDGFRα promoter region, rs6554162 (−1309A/G) and rs1800812 (−635G/T), have been significantly associated with increased susceptibility to PTC, as demonstrated in a Korean cohort. Individuals harboring the risk alleles (A at rs6554162 or T at rs1800812) were disproportionately represented among PTC patients, suggesting these variants may enhance PDGFRα transcriptional activity or receptor function, thereby promoting oncogenic signaling cascades.^20-22^ PDGFRα overexpression identified in tumor cells correlates with aggressive features such as lymph node metastasis and recurrence, supporting activation of downstream cascades including the RAS–MAPK/ERK and PI3K/AKT pathways, which have been extensively documented in thyroid tumorigenesis.^13,15,25^ Mechanistic studies have shown that PDGFRα ligand binding induces receptor dimerization and autophosphorylation, triggering the recruitment of adaptor proteins that activate these pathways, promoting cell proliferation, migration, and survival.^12,13,30^ The activation of MAPK/ERK signaling by PDGFRα leads to transcriptional upregulation of genes driving cell cycle progression and invasion, while PI3K/AKT activation enhances cellular metabolism and resistance to apoptosis.^13,15,31^ Importantly, recent experimental evidence has demonstrated that PDGFRα activation suppresses thyroid differentiation markers, including the sodium-iodide symporter (NIS), through downregulation of thyroid transcription factors such as TTF-1.^12,15,32^ This mechanistic link explains the clinical observation from our review that PDGFRα overexpression is associated with RAI therapy resistance. Such functional interference with thyroid-specific gene expression establishes PDGFRα not only as a driver of tumor aggressiveness but also as a mediator of treatment failure, highlighting its dual oncogenic role in PTC.^12,15,20^

 PDGFRβ’s molecular function in thyroid cancer predominantly involves modulating the tumor microenvironment rather than direct oncogenic signaling within tumor cells. Our findings align with a growing body of evidence demonstrating that PDGFRβ expressed on cancer-associated fibroblasts (CAFs) and pericytes plays a central role in remodeling the tumor stroma and promoting angiogenesis.^26,27,29,32^ Activation of PDGFRβ by tumor-derived PDGF-BB stimulates fibroblast proliferation and differentiation into CAFs, which secrete extracellular matrix components and growth factors, reinforcing a desmoplastic and immunosuppressive microenvironment.^13,27^ Concurrently, PDGFRβ signaling in pericytes stabilizes neovasculature critical for tumor growth and metastatic dissemination. At the signaling level, PDGFRβ engagement triggers MAPK/ERK and PI3K/AKT pathways in stromal cells, paralleling the tumor cell signaling axis but functioning to maintain stromal cell survival and pro-tumorigenic activity.^12,27^ The ability of multikinase inhibitors such as lenvatinib to disrupt PDGFRβ-mediated pericyte function and thereby impair tumor vasculature elucidates the clinical efficacy observed in advanced thyroid cancer treatment. Thus, PDGFRβ contributes to tumor progression by shaping the tumor stroma and maintaining blood vessels that support tumor growth.^23,27,33,34^ The differential compartmentalization of PDGFRα and PDGFRβ signaling underscores their complementary molecular roles, PDGFRα directly drives tumor cell proliferation and dedifferentiation, while PDGFRβ regulates the tumor-supportive microenvironment through stromal cell activation and angiogenesis.^13,15,27,35^

 Our review confirms that elevated PDGFRα expression is a consistent feature in PTC, paralleling findings in other solid tumors. For example, PDGFRα copy-number gains in uterine corpus endometrial carcinoma associate with poorer overall survival, similar to PDGFRα’s link to lymph node metastasis and recurrence in PTC.^21,22,36^ In hepatocellular carcinoma, PDGFRα overexpression correlates with increased angiogenesis and worse prognosis, underscoring its conserved role in tumor proliferation and invasiveness across epithelial cancers.^31^ Mechanistically, PDGFRα activates MAPK/ERK and PI3K/Akt pathways, consistent with PTC data showing PDGFRα signaling reduces sodium iodide symporter expression, promoting RAI resistance.^17,37^ Genetic studies highlight promoter SNPs and copy-number variations influencing PDGFRα expression and tumor behavior in PTC.^20-22^ PDGFRα positivity shows high sensitivity (86%) and specificity (71%) for lymph node metastasis in PTC, reinforcing its prognostic value. Conversely, PDGFRβ expression varies by tumor lineage and stromal context; for instance, high PDGFRβ is linked to stromal activation and poor outcomes in ovarian cancer, mirroring its context-dependent associations in thyroid cancer.^38,39^ Collectively, these findings support PDGFRα as a tumor-intrinsic driver of proliferation and invasiveness, while PDGFRβ’s prognostic role is largely shaped by the tumor microenvironment. Integrating genomic, proteomic, and clinical data across cancers strengthens the rationale for PDGFR profiling to improve thyroid cancer risk stratification and therapeutic strategies.

 Our review found that elevated PDGFRα expression in PTC generally associates with worse disease-free survival and higher recurrence, although some variability exists among studies. In glioblastoma, PDGFRα copy-number gains consistently predict reduced overall survival,^40^ and pan-cancer analyses identify PDGFRα-pathway copy-number gain as an independent risk factor for shorter survival in several malignancies.^36^ Aberrant PDGFRα expression in HER2-positive breast cancer models also correlates with aggressive, treatment-resistant phenotypes.^41^ High intratumoral PDGFRβ-positive fibroblast density has been linked to increased risk of death and disease progression in multiple cancers, including breast, pancreatic, and lung cancers.^36,42^ Stromal PDGFRβ expression in breast cancer brain metastases predicts shorter recurrence-free survival, illustrating its role in the tumor microenvironment and metastatic progression.^43^ The dual prognostic roles of PDGFRβ reflect its function as a stromal activation marker that can either promote or restrain tumor progression depending on context.^43,44^ These findings support incorporating PDGFRα and PDGFRβ into thyroid cancer risk stratification panels and provide rationale for PDGFR-targeted therapies. Preclinical data in PTC demonstrate that sunitinib inhibits PDGFRα signaling and may overcome RAI resistance, suggesting potential benefit from combination therapies stratified by receptor status in future clinical trials.

 Targeting PDGFRα/β has shown significant therapeutic success in other cancers, such as the NAVIGATOR trial where avapritinib achieved an 88% response rate in PDGFRα-mutant gastrointestinal stromal tumors (GIST) by inhibiting resistance mutations. Next-generation inhibitors like ripretinib, avapritinib, and crenolanib demonstrate mutation-specific efficacy, highlighting the importance of tailoring therapy to genetic context.^45,46^ Similarly, in PTC, PDGFRα overexpression contributes to RAI resistance by reducing sodium-iodide symporter levels, requiring higher RAI doses.^15^ Sunitinib effectively inhibits PDGFRα-driven signaling pathways (MAPK/ERK, PI3K/Akt) and suppresses tumor cell proliferation in PTC models.^13^ Studies in other malignancies reveal that receptor tyrosine kinase cross-talk and compensatory upregulation of PDGFRα/β can drive resistance to targeted therapies, suggesting combination treatments may be necessary to overcome adaptive resistance.^34,47^ In PTC, high PDGFRα expression correlates with poorer disease-free survival and lymph node metastasis, supporting mutation-guided tyrosine kinase inhibitor strategies.^13,24^ Ligand-induced PDGFRα/β activation stimulates downstream pathways (JAK/STAT, Notch) involved in tumor progression, offering multiple intervention points.^48,49^ Preclinical data reinforce the potential of multi-kinase inhibitors in iodine-refractory PTC.^13^ However, experiences from GIST caution that resistance mutations can limit inhibitor effectiveness, necessitating monitoring of tumor evolution.^50^ Additionally, intracellular regulators such as PDZK1 modulate PDGFRβ signaling and may serve as novel therapeutic targets.^51^ The concept of receptor plasticity further supports adaptive or sequential combination therapies to prevent resistance in PTC.^47,52^ Therapeutic targeting of PDGFRα/β in thyroid cancer holds promise through mutation-specific inhibitors, combination therapies addressing compensatory pathways, stromal modulation, and resistance monitoring—strategies informed by both PTC-focused data and broader oncology experiences.

## Limitations

 Our review is limited by significant heterogeneity across the included studies, including variable immunohistochemically scoring systems, diverse assay platforms (IHC, qPCR, Western blot), and inconsistent cut-off definitions for PDGFRs positivity, complicating direct comparison and meta-analysis. Many cohorts were small, reducing statistical power and increasing the risk of type II error. The predominance of retrospective, single‐center designs also introduce selection bias and limit the generalizability of our findings to broader patient populations. Furthermore, most studies lacked comprehensive molecular annotation beyond BRAF and RET status, precluding more nuanced analyses of PDGFR expression in the context of other genetic drivers. Publication bias may have inflated observed associations; as negative or null studies are less likely to be reported. Finally, the absence of longitudinal data on PDGFR dynamics over the disease course restricts our ability to assess temporal changes in receptor expression and their relationship to treatment response or resistance.

## Conclusion

 In conclusion, this systematic review suggests that PDGFRα may be a potential biomarker of aggressiveness and RAI resistance in PTC, with consistent associations to lymph node metastasis, larger tumor size, and poorer disease-free survival. PDGFRβ exhibits a more context-dependent role, predominantly reflecting stromal activation and angiogenesis, and warrants further investigation to clarify its prognostic and therapeutic value. However, before clinical implementation, standardized assay protocols, prospective validation in multicenter cohorts, and incorporation of comprehensive molecular profiling are essential. Future studies are needed to also explore the efficacy of PDGFRα inhibition alongside conventional treatments to enhance RAI uptake. Ultimately, integrating PDGFRs assessment into thyroid cancer management may improve risk stratification and pave the way for more personalized, effective therapeutic strategies.

## Future Perspectives

 Emerging evidence suggests that rare PDGFR genetic alterations may define distinct biological subgroups in thyroid and other solid tumors. For instance, PDGFRα copy-number gains have been reported in a subset of anaplastic thyroid carcinoma (ATC), where they may co-occur with other receptor tyrosine kinase amplifications and potentially indicate a dedifferentiated phenotype with therapeutic implications. In addition, germline PDGFRα promoter polymorphisms have been associated with increased susceptibility to PTC, while other variants identified in less aggressive tumors, such as CASTLE, may reflect more indolent biological behavior. In contrast, germline variations in PDGFRβ have not shown consistent associations, suggesting that its role may be more related to stromal expression rather than inherited genetic changes.

 Although these findings are outside the scope of the present systematic review, but highlight the potential prognostic and therapeutic relevance of PDGFR alterations and further investigation in future studies.

## Availability of Data and Materials

 The dataset of this work is available from corresponding author upon request.

## Competing Interests

 The authors announce that have no conflicts of interest.

## Consent for Publication

 Notapplicable.

## Ethical Approval

 This study is affiliated with the Iran University of Medical Sciences,

 where it has received ethical approval: IR.IUMS.REC. 1403.1119.

## Supplementary File

Supplementary data for Search strategy and the REMARK criteria for all included studies.
