# Supplementary material for: Molecular and Clinical Significance of PDGFR Family in Papillary Thyroid Cancer: A Systematic Review
Source: Adv Pharm Bull. 2026 May 24;16(3):454–62. doi: 10.34172/apb.46975 (PMC13745378; doi:10.34172/apb.46975)
Supplement: Supplementary file 1 [file apb-16-454-s001.pdf]

Adv Pharm Bull 2026;16(3):454-S1
doi:10.34172/apb.46975
https://apb.tbzmed.ac.ir

Table S1. Search strategy in the systematic review

	Databases	Search Strategy	Finding
**Search Strategy for Expression Studies**	**pubmed**	(((Cancer*) OR (Neoplasm*) OR (Carcinoma*) OR (Adenoma*) OR (Nodule*)) AND (Thyroid*)) AND ((PDGFR*) OR (Platelet Derived Growth Factor receptor*) OR (CD140*))	139
	**Web of science**	(((Cancer*) OR (Neoplasm*) OR (Carcinoma*) OR (Adenoma*) OR (Nodule*)) AND (Thyroid*)) AND ((PDGFR*) OR (Platelet Derived Growth Factor receptor*) OR (CD140*))	179
	**scopus**	(((Cancer*) OR (Neoplasm*) OR (Carcinoma*) OR (Adenoma*) OR (Nodule*)) AND (Thyroid*)) AND ((PDGFR*) OR (Platelet Derived Growth Factor receptor*) OR (CD140*))	533
**Search Strategy For Genetic Alternation Studies**	**pubmed**	((((((Neoplasm*[Title/Abstract]) OR (Carcinoma*[Title/Abstract])) OR (Adenoma*[Title/Abstract])) OR (Nodule*[Title/Abstract])) AND (Thyroid*[Title/Abstract])) AND (((PDGFR*[Title/Abstract]) OR (Platelet Derived Growth Factor receptor*[Title/Abstract])) OR (CD140*[Title/Abstract]))) AND (((((((((((((((((Polymorphisms, Genetic[Title/Abstract]) OR (Gene Polymorphism[Title/Abstract])) OR (Gene Polymorphisms[Title/Abstract])) OR (Polymorphism, Gene[Title/Abstract])) OR (Polymorphisms, Gene[Title/Abstract])) OR (Polymorphism[Title/Abstract])) OR (Genetics[Title/Abstract])) OR (Polymorphisms[Title/Abstract])) OR (Genetics[Title/Abstract])) OR (Genetic Polymorphisms[Title/Abstract])) OR (Genetic Polymorphism[Title/Abstract])) OR (Genetic Variations[Title/Abstract])) OR (Variations, Genetic[Title/Abstract])) OR (Diversity, Genetic[Title/Abstract])) OR (Diversities, Genetic[Title/Abstract])) OR (Genetic Diversities[Title/Abstract])) OR (Genetic Diversity[Title/Abstract])) OR (Variation, Genetic[Title/Abstract]))	5
	**Web of science**	TS=(((((((Neoplasm*) OR (Carcinoma*)) OR (Adenoma*)) OR (Nodule*)) AND (Thyroid*)) AND (((PDGFR*) OR (Platelet Derived Growth Factor receptor*)) OR (CD140*))) AND (((((((((((((((((Polymorphisms, Genetic) OR (Gene Polymorphism)) OR (Gene Polymorphisms)) OR (Polymorphism, Gene)) OR (Polymorphisms, Gene)) OR (Polymorphism)) OR (Genetics)) OR (Polymorphisms)) OR (Genetics)) OR (Genetic Polymorphisms)) OR (Genetic Polymorphism)) OR (Genetic Variations)) OR (Variations, Genetic)) OR (Diversity, Genetic)) OR (Diversities, Genetic)) OR (Genetic Diversities)) OR (Genetic Diversity)) OR (Variation, Genetic)))	9
	**scopus**	KEY (((((((neoplasm*) OR (carcinoma*)) OR (adenoma*)) OR (nodule*)) AND (thyroid*)) AND (((pdgfr*) OR (platelet AND derived AND growth AND factor AND receptor*)) OR (cd140*))) AND (((((((((((((((((polymorphisms, AND genetic) OR (gene AND polymorphism)) OR (gene AND polymorphisms)) OR (polymorphism, AND gene)) OR (polymorphisms, AND gene)) OR (polymorphism)) OR (genetics)) OR (polymorphisms)) OR (genetics)) OR (genetic AND polymorphisms)) OR (g enetic AND polymorphism)) OR (genetic AND variations)) OR (variations, AND genetic)) OR (diversity, AND genetic)) OR (div ersities, AND genetic)) OR (genetic AND diversities)) OR (genetic AND diversity)) OR (variation, AND genetic)))	121

Table S2. The Reporting Recommendations for Tumor Marker Prognostic Studies (REMARK) criteria for all included studies

		Genetic Alternation Studies			Expression Studies								
**REMARK Criteria**	**Study**	Cruz-Romero et al. (2024)[20]	Borowczyk et al. (2019)[21]	Kim et al. (2012)[22]	Zhu et al. (2023)[23]	Shi et al. (2020)[24]	Adewuyi et al. (2018)[25]	Sun et al. (2016)[26]	Campistrous et al. (2016)[15]	Lee et al. (2016)[27]	Zhang et al. (2012)[13]	Durante et al. (2011)[28]	Elliott et al. (2008)[29]
**INTRODUCTION**	1.*Introduction*	1	0.5	1	1	1	1	1	1	1	1	1	1
**MATERIALS AND METHODS**	2.*Patients*	1	1	1	0.5	1	0.5	1	1	1	1	1	1
	3.*Specimen characteristics*	1	0	0	0	0	0	0	1	0	0	0	0
	4.*Assay methods*	1	1	1	1	1	1	1	1	1	1	1	1
	5.*Study design*	1	1	1	1	1	1	1	1	1	1	1	1
	6. Type of study, stratification/matching, time period and follow up	1	1	1	1	0	0.5	1	1	1	1	1	1
	7. Clinical endpoints	0	0	0.5	1	0	0	1	1	0	0	0	1
	8. Variables examined	1	0.5	1	1	1	1	1	1	1	1	1	1
	9. Sample size rationale	1	1	1	1	1	1	1	1	1	1	1	1
	10. Statistical methods	1	1	1	1	1	1	1	1	1	1	1	0
	11. How marker values were handled	1	1	1	1	1	1	1	1	1	1	1	1
**RESULTS**	*12. All patients accounted*	0.5	1	1	1	1	1	1	1	1	1	1	1
	13. Descriptive statistics	1	1	1	1	1	1	1	1	1	1	1	1
	14. Reaction of marker to prognostic variable	0	0	1	0	1	1	1	1	1	1	0	0
	15. Univariate analysis	0	0	0	0	1	1	1	1	1	1	1	0
	16. Multivariate analysis	0.5	0	1	1	0	0	1	0	0	0	0	0
	17. Estimated effects	0	0.5	1	0	1	1	1	0	0	1	0	0
	18. Further investigations	0	0	1	1	0	0	0	1	1	1	1	0
**DISCUSSION**	*19. Interpretation of results*	1	1	1	1	1	1	1	1	1	1	1	1
	20. Future directions	0.5	0.5	1	1	1	1	1	1	1	1	1	1
**Total REMARK Score**		14	12	18	16	15	15	18	18	16	17	15	13
